# Advanced XRD peak broadening analysis of gallium-doped ZnO nanoparticles for crystallite size evaluation

**DOI:** 10.1038/s41598-025-31317-2

**Published:** 2025-12-10

**Authors:** Ali Khorsand Zak, Abd Manaf Hashim

**Affiliations:** 1 Nanobiotechnology Lab, Central Lab, Esfarayen University of Technology, Esfarayen, 96619-98195 North Khorasan Iran; 2 Malaysia-Japan International Institute of Technology (MJIIT), Universiti Teknologi Malaysia (UTM), Jalan Sultan Yahya Petra, 54100 Kuala Lumpur, Malaysia

**Keywords:** ZnO, Zinc oxide, Gallium doped ZnO, XRD, Peak broadening, Chemistry, Materials science, Nanoscience and technology

## Abstract

This study investigates the most reliable approach for determining the crystallite size of nanoparticles through advanced X-ray diffraction (XRD) analysis. Gallium-doped zinc oxide nanoparticles (ZnO-NPs) were synthesized via a gelatin-assisted sol–gel method with Ga concentrations of 0%, 3%, 9%, and 15%. Structural characterization was performed using XRD, and crystallite size and lattice strain were evaluated through multiple analytical methods, including Scherrer’s equation, the Williamson–Hall (W–H) method, and both simple and modified Size–Strain Plot (SSP and MSSP) techniques. These complementary approaches enabled clear separation of size and strain effects, offering deeper insight into the microstructural evolution with increasing Ga content. The results revealed that higher Ga doping led to smaller crystallite sizes and increased lattice strain. Overall, the findings demonstrate that the MSSP method provides superior accuracy for nanoscale material characterization and highlight the significant influence of Ga incorporation on the structural properties of ZnO nanoparticles.

## Introduction

Zinc oxide (ZnO) is a multifunctional semiconductor that has garnered considerable attention due to its unique combination of physical and chemical properties, including a wide direct bandgap (~ 3.32 eV), high exciton binding energy (~ 60 meV), and strong thermal and chemical stability^1^. These characteristics make ZnO an attractive material for a variety of applications such as optoelectronics, piezoelectric devices, gas sensors, photocatalysis, and biomedical fields^2–7^. Among the various strategies to tailor the properties of ZnO nanomaterials (ZnO-NPs) by doping with various elements from the periodic table, doping with group III elements such as gallium (Ga), aluminum (Al), and indium (In) has shown remarkable effectiveness^8–11^. Gallium, in particular, is known for its ability to substitute Zn^2^⁺ ions in the ZnO lattice due to its comparable ionic radius and higher valence state, leading to changes in carrier concentration, crystallite size, and microstructural strain. Ga doping can enhance electrical conductivity, modify optical transparency, and introduce controlled lattice distortions that improve or redirect the performance of ZnO-based systems for specific applications^12,13^. For example, smaller crystallite size and higher lattice strain significantly influence the functional properties of Ga-doped ZnO. The reduction in crystallite size increases the density of grain boundaries, which can scatter charge carriers and slightly lower their mobility; however, Ga doping simultaneously increases carrier concentration, often compensating for this effect and resulting in improved overall conductivity^14^. The presence of lattice strain introduces defects such as oxygen vacancies and interstitials, which act as donor states, further enhancing electrical conductivity. From a photocatalytic perspective, smaller crystallites provide a larger surface-to-volume ratio and more active sites for catalytic reactions, while the strain-induced defects facilitate charge separation by trapping photogenerated carriers and suppressing their recombination^15^. Together, these effects lead to enhanced light absorption, improved carrier dynamics, and superior photocatalytic efficiency.

While X-ray diffraction (XRD) remains a fundamental tool for analyzing crystalline phases and estimating crystallite sizes, it faces limitations in deconvoluting the effects of strain and size-induced peak broadening, especially in doped or nanostructured systems^16,17^. Traditional Scherrer-based analysis provides a basic estimation of crystallite size but neglects lattice strain and instrumental effects, often leading to oversimplified interpretations^18,19^. The Williamson–Hall (W–H) approach employs three distinct analytical models: the Uniform Deformation Model (UDM), the Uniform Stress Deformation Model (USDM), and the Uniform Deformation Energy Density Model (UDEDM)^20^. These models are utilized to evaluate different aspects of lattice strain and elastic behavior in crystalline materials. In comparison, the Size–Strain Plot (SSP) technique assumes that strain-induced broadening follows a Gaussian distribution, whereas size-related broadening is best represented by a Lorentzian profile for the diffraction peaks^21^. To overcome these drawbacks, more advanced models such as the Williamson–Hall (W–H) and Size-Strain Plot (SSP) methods are employed^18^. These allow for simultaneous estimation of crystallite size and microstrain, offering a more accurate understanding of structural distortions introduced by Ga doping.

Although, several different methods have been used to synthesis ZnO-NPs, including sol–gel, sonochemical, combustion, solvothermal, precipitations, laser ablation, and CVD, synthesis of high-quality, doped requires a controlled and reproducible method to ensure uniformity in particle size, morphology, and dopant distribution^22–30^. The sol–gel method offers several advantages over conventional synthesis techniques such as solid-state reactions or hydrothermal methods. It enables molecular-level mixing of precursors, which ensures excellent chemical homogeneity and high-purity final products^31,32^. This process typically requires lower calcination and sintering temperatures, making it more energy-efficient and ideal for preserving nanoscale features. Additionally, the sol–gel route provides precise control over composition and allows the production of ultrafine, uniformly distributed nanoparticles^33,34^. Its versatility also enables the fabrication of various forms, including powders, thin films, coatings, and monoliths, from the same precursor solution^35,36^.

This study focuses on the synthesis of Ga-doped ZnO nanoparticles using a gelatin-assisted sol–gel route and systematically investigates the influence of Ga content on the structural and morphological properties of the resulting nanomaterials. Samples with different Ga doping concentrations (0%, 3%, 9%, and 15%) were synthesized and subjected to detailed characterization using XRD and TEM. Crystallite sizes and lattice strains were calculated using Scherrer, Williamson–Hall, and SSP methods to evaluate the accuracy and consistency of each technique. Many relationships have been established in the past using the SSP method, but we conducted a careful study to obtain the best relationship for calculating crystal size until we reached the final and correct relationship. The goal is to elucidate the role of Ga doping in tailoring ZnO’s nanoscale structure and to validate the use of advanced diffraction analysis techniques for accurate microstructural evaluation.

## Materials and methods

### Materials

The initial quantities of gallium nitrate (Ga(NO_3_)_3_, 99% from Sigms-Aldrich) and zinc nitrates hexahydrate (Zn(NO_3_)_2_.6H_2_O purchased from Sigma-Aldrich, 99%) were used as the precursors materials, gelatin (Type B from bovine, Sigma-Aldrich) was used as polymeric agent, and distilled water, DW, was used as solvent in the reactions.

### Synthesis procedure

It was targeted to obtain 2 g of the final product and the value of the precursors were measured and calculated based on Eq. (1) reaction were the values of x = 0.0, 0.03, 0.09, and 0.15 referred to ZnO, ZG3, ZG9, and ZG15, respectively.

$$\left(1-x\right)Zn{\left({NO}_{3}\right)}_{2}\bullet 6{H}_{2}O + xGa{\left({NO}_{3}\right)}_{3}\stackrel{\text{gelatin and calcinations}}{\to }$$

1 $${Zn}_{1-x}{Ga}_{x}O + \left(A\right){NO}_{2}+\left(B\right){CO}_{2}+\left(C\right){H}_{2}O$$

where, (A), (B), and (C) are uncertain constant depend on gelatin value.

**Fig. 1:**
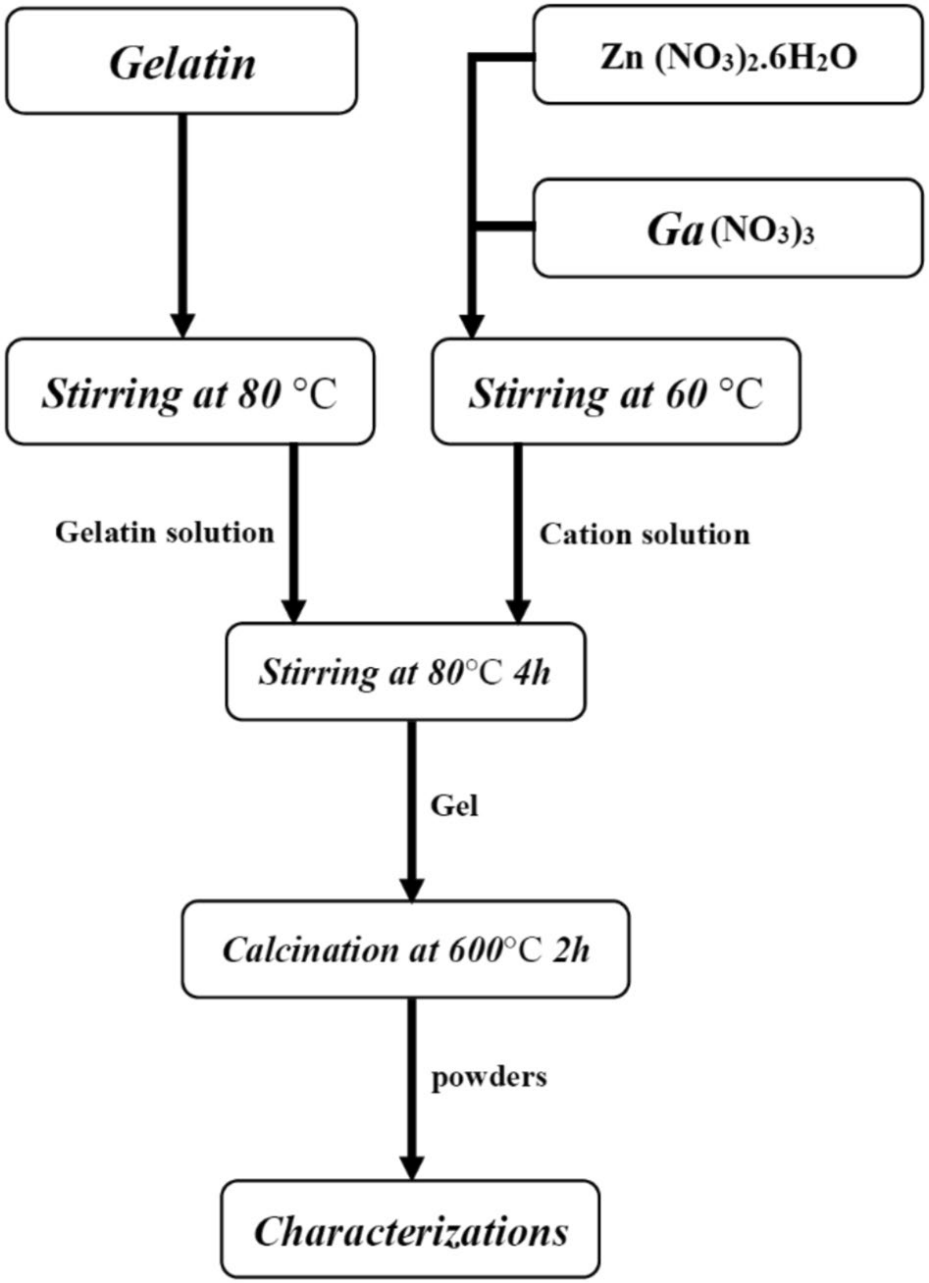
The synthesis procedure flowchart.

The required quantities of precursor materials were calculated according to the desired final composition. Gelatin was used as a complexing agent in an amount approximately twice the weight of the target product. An oil bath system was employed to ensure stable and uniform heating throughout the process. A glass cylindrical container with a capacity of 100–150 mL was placed in the oil bath, allowing free rotation of the magnetic stirrer positioned at the bottom. Alternatively, a mechanical stirrer could be used to achieve effective mixing. Approximately 80 mL of distilled water was added to the container, and the oil bath temperature was maintained at 80 °C. The calculated amounts of metal nitrate salts were gradually introduced into the water and stirred at 150 rpm until completely dissolved, yielding a clear and homogeneous solution. Gelatin powder was then added slowly under continuous stirring to ensure proper dispersion. After a short period, a transparent yellow solution was obtained. The stirring speed was subsequently reduced, and heating was continued until a thick brown gel with a honey-like consistency formed. The resulting gel was carefully spread along the inner surface of an alumina crucible and calcined in air at 600 °C for 2 h, producing a uniform white powder. This synthesis procedure was repeated for all samples with varying gallium concentrations. The resulting powders were collected and subjected to further structural and microstructural characterization, Fig. 1.

### Characterizations

X-ray diffraction (XRD) analysis of the synthesized powders was performed using a Philips D5000 diffractometer equipped with Cu Kα radiation (λ = 1.5406 Å). Diffraction patterns were recorded over a 2θ range of 20°–70° with a step size of 0.05° and a counting time of 35 s per step. The crystallite size and lattice strain were evaluated using the Williamson–Hall (W–H) and Size–Strain Plot (SSP) methods to separate the contributions of size broadening and microstrain. The particle morphology was examined by transmission electron microscopy (TEM) using a Hitachi H-7100 microscope. TEM micrographs were subsequently analyzed with Digimizer software to determine the particle size distribution.

## Results and discussions

### XRD analyzes of pure and Ga-doped ZnO nanoparticles

**Fig.2:**
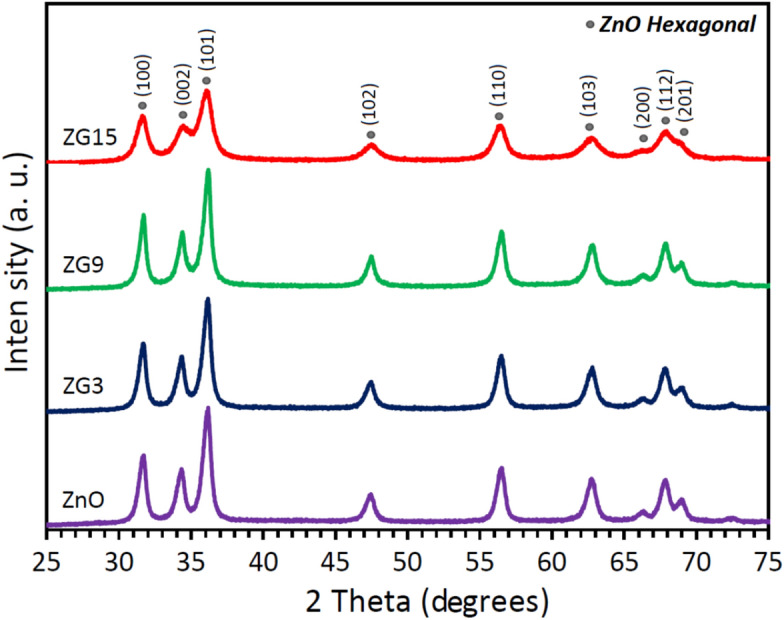
XRD patterns of the prepared nanoparticle with different value of Ga.

**Fig. 3:**
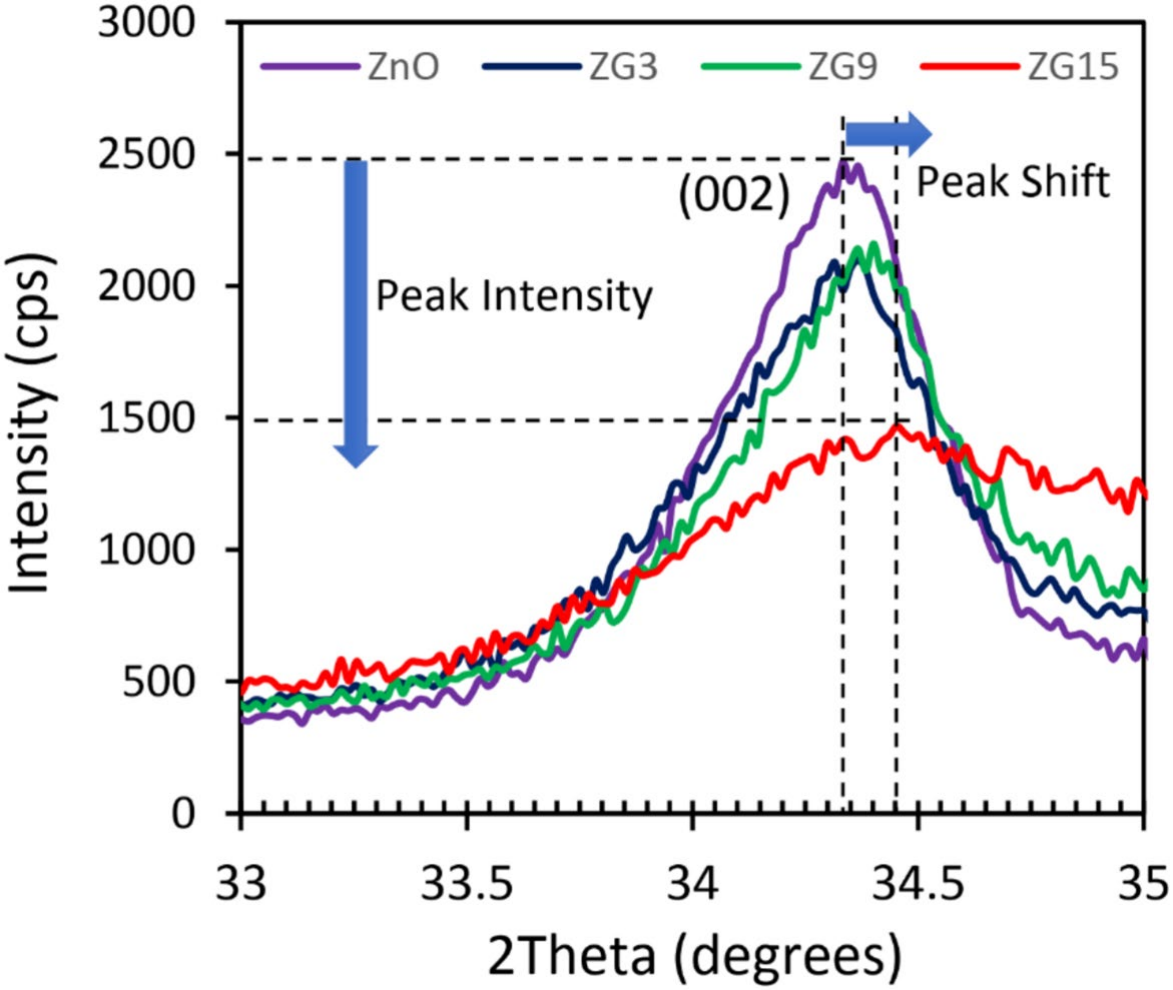
(002) diffraction peak related to the pure and doped samples. It is clearly seen both intensity and position of the peak variated by Ga volume.

Figure 2 present the XRD patterns of the prepared pure and Ga-doped ZnO nanoparticles. The results indicated that Ga atoms diffused in ZnO lattice strain and some of the Zn atoms have been substituted by Ga atoms. Also, in our previous work the XPS results proved the existence of Ga atoms in the composite^8^. Therefore all of the detected peaks are attributed to the hexagonal ZnO lattice and there is no other peaks related to the other compounds that include Ga, (PDF NO: 00–005-0664, space group P63mc, number 186). It is evident that incorporating Ga into the ZnO lattice causes significant peak broadening, along with a decrease in the intensity of the diffraction peaks. Additionally, the diffraction peaks shift toward higher angles, which is attributed to lattice distortion and a reduction in lattice parameters due to the substitution of smaller Ga^3^⁺ ions for Zn^2^⁺ ions. The observed peak broadening indicates a decrease in crystallite size and an increase in microstrain within the ZnO lattice, which can be further analyzed using the Williamson–Hall method to quantify the contributions of size and strain effects, Fig. 3.

The Scherrer equation is a fundamental formula in x-ray diffraction (XRD) analysis to estimate the average size of crystallites in materials^37^. Most researchers used this equation for the calculation of the crystallite size but it would be also helpful to fallow its fundamental and source. The Scherrer equation is derived from the principles of x-ray diffraction and were interfaces. Bragg law is the most famous relation in crystallography that describes the condition for constructive interference x-ray scattered by crystal planes as Eq. (2).

2 $$2d\mathrm{sin}\theta =n\lambda$$

where d is the inter-planer spacing, θ is the Bragg angle, λ is the wavelength, and n is the order of diffraction.

For a finite size crystal D thickness is in the direction of the diffraction vector^38^. Therefore, the number of the contributing lattice planes is $N=\frac{D}{d}$ and the destructive interface occurs when the phase diffraction between waves reflected from the first and lost planes reaches π. A small deviation Δθ from the Bragg angle θ introduces a cumulative phase difference occurs the crystallite and presented by Eq. (3)^39^.

3 $$\Delta \varphi =2\pi N\bullet \frac{\Delta d}{d}$$

where Δd is the effective lattice spacing variation due to the angular derivation. As mentioned earlier, when $\Delta \varphi =\pi$, for destructive interference at the peaks we have:

$$\Delta \varphi =\pi \stackrel{Eq. 3}{\Rightarrow }N\bullet \frac{\Delta d}{d}=\frac{1}{2} \stackrel{ N=\frac{D}{d} }{\to } \frac{D}{d}\bullet \frac{\Delta d}{d}=\frac{1}{2}\stackrel{}{\Rightarrow }\Delta d=\frac{{d}^{2}}{2D}$$

Now we back to Bragg relation (λ = 2dsinθ) and calculate its derivative with respect to θ below:

$$0=2\frac{\Delta d}{\Delta \theta }\mathrm{sin}\theta +2d\mathrm{cos}\theta \bullet \Delta \theta \stackrel{}{\Rightarrow } \frac{\Delta d}{\Delta \theta }\mathrm{sin}\theta =-d\mathrm{cos}\theta \bullet \Delta \theta \stackrel{}{\Rightarrow }\Delta \theta =-\frac{\Delta d}{d}\mathrm{tan}\theta$$

Δd substituted by $\frac{{d}^{2}}{2D}$ which was obtained earlier.

$$\Delta \theta =-\frac{{d}^{2}}{2D}\mathrm{tan}\theta \underset{}{\Rightarrow }\left|\Delta \theta \right|=\frac{{d}^{2}}{2D}\mathrm{tan}\theta =\frac{{d}^{2}\mathrm{sin}\theta }{2D\mathrm{cos}\theta }$$

But we know that, the total angular spread FWHM (β) is approximately twice the deviation $\left|\Delta \theta \right|$ and therefore the Scherrer equation is derived as fallow:

$$\beta \cong 2\left|\Delta \theta \right|=\frac{d\mathrm{sin}\theta }{D\mathrm{cos}\theta }=\frac{\raisebox{1ex}{$\lambda $}\!\left/ \!\raisebox{-1ex}{$2$}\right.}{D\mathrm{cos}\theta }\stackrel{}{\Rightarrow }D=\frac{\lambda }{\beta \mathrm{cos}\theta }\bullet \frac{1}{2}$$

And finally:

4 $$D=\frac{K\lambda }{\beta \mathrm{cos}\theta }$$

where K is a constant known as shape factor, D is the average crystallite size, β is FWHM in radian, λ is the wavelength, and θ if the Bragge angle corresponding to the diffraction peak.

The Scherrer, Eq. (4), method typically assumes crystallites are spherical or cubic for simplicity, with the Scherrer constant (K) adjusted accordingly K≈0.89–0.91 for spheres or cubes, while anisotropic shapes like plates or rods require higher K values (~ 1.0–1.2). The calculated size (D) corresponds to the dimension perpendicular to the diffracting planes, meaning plate-like crystallites report thickness, not lateral size^40^. For practical use, K = 0.9 is a common default unless the material’s crystallite geometry is well-characterized, though accuracy diminishes for irregular or aggregated shapes, necessitating validation via microscopy (TEM/SEM) or advanced methods like Rietveld refinement for complex morphologies.

#### Crystallite size of Ga-doped ZnO-NPs using Scherrer method

In this method the data of the main peak, (101), are taken to use Eq. (4) and calculating the crystallite sizes. The results are obtained to be 26, 24, 22, and 13 nm For ZnO, ZG3, ZG9, and ZG15, respectively.

The problem is, this equation assumes peak broadening is solely due to crystallite size ignoring contribution from lattice strain or instrumental effects^41^. Then results represents an average overs all crystallites contributing to the diffraction peak. The most intense peak mast be considered. Also, this method is suitable for isotropic particles with size smaller than 100 nm. To have more accurate results by using Scherrer method.

**Fig. 4:**
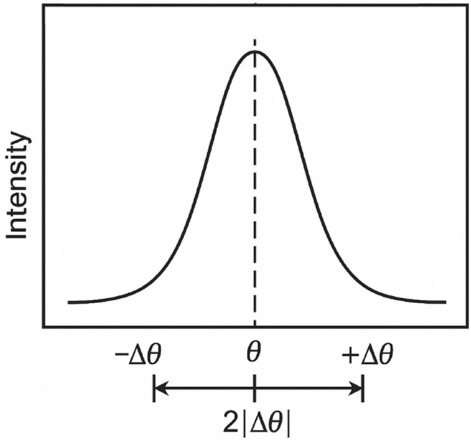
Symmetrical broadening of an XRD peak showing angular spread from –Δθ to + Δθ, with the total broadening represented as 2|Δθ|.

For materials with strain broadening the Scherrer equation mast be combined with strain term. We obtained earlier $\left|\Delta \theta \right|=\frac{\Delta d}{d}\mathrm{tan}\theta$ and β_hkl_ accounts for symmetrical broadening on both side of the diffraction peak from –Δθ to + Δθ, therefore, the total angular spread is $2\left|\Delta \theta \right|$, Fig. 4, however, in practice the broadening is empirically observed to scale as Eq. (5)^42^.

5 $${\beta }_{strain}=4\varepsilon \mathrm{tan}\theta$$

The constant is 4 because the strain induced angular spread affects both sides of the peak (doubling $\left|\Delta \theta \right|$)^43^. In diffraction real materials have a distribution of strains (Gaussian or Lorentzian). Where FWHM relates to the standard deviation σε^18^. For Gaussian profiles FWHM ≈ 4σε leading to ${\beta }_{strain}=4\varepsilon \mathrm{tan}\theta$^42^. At higher angles (θ) small strain variations cause larger angular broadening from due to the tanθ term. It can be contributed that factor 4 is obtained from combines contribution form, symmetrical angular spread and strain distribution effects, Eq. (6). Know the Scherrer equation is modified as below^18^:

6 $${\beta }_{hkl}={\beta }_{size}+{\beta }_{strain}\underset{}{\Rightarrow }$$

$${\beta }_{hkl}=\frac{K\lambda }{D\mathrm{cos}\theta }+4\varepsilon \mathrm{tan}\theta \underset{}{\Rightarrow }$$

7 $${\beta }_{hkl}\mathrm{cos}\theta =\frac{K\lambda }{D}+4\varepsilon \mathrm{sin}\theta$$

The term βcosθ is plotted with respect to 4sinθ. The average crystallite size (D) is obtained from intersection of the line with the vertical axis and lattice strain from the sloop of the linearly fitted data. This method known as Williamson–Hall method, Eq. (7)^44,45^.

#### Crystallite size of Ga-doped ZnO-NPs using Williamson–Hall method

**Fig. 5:**
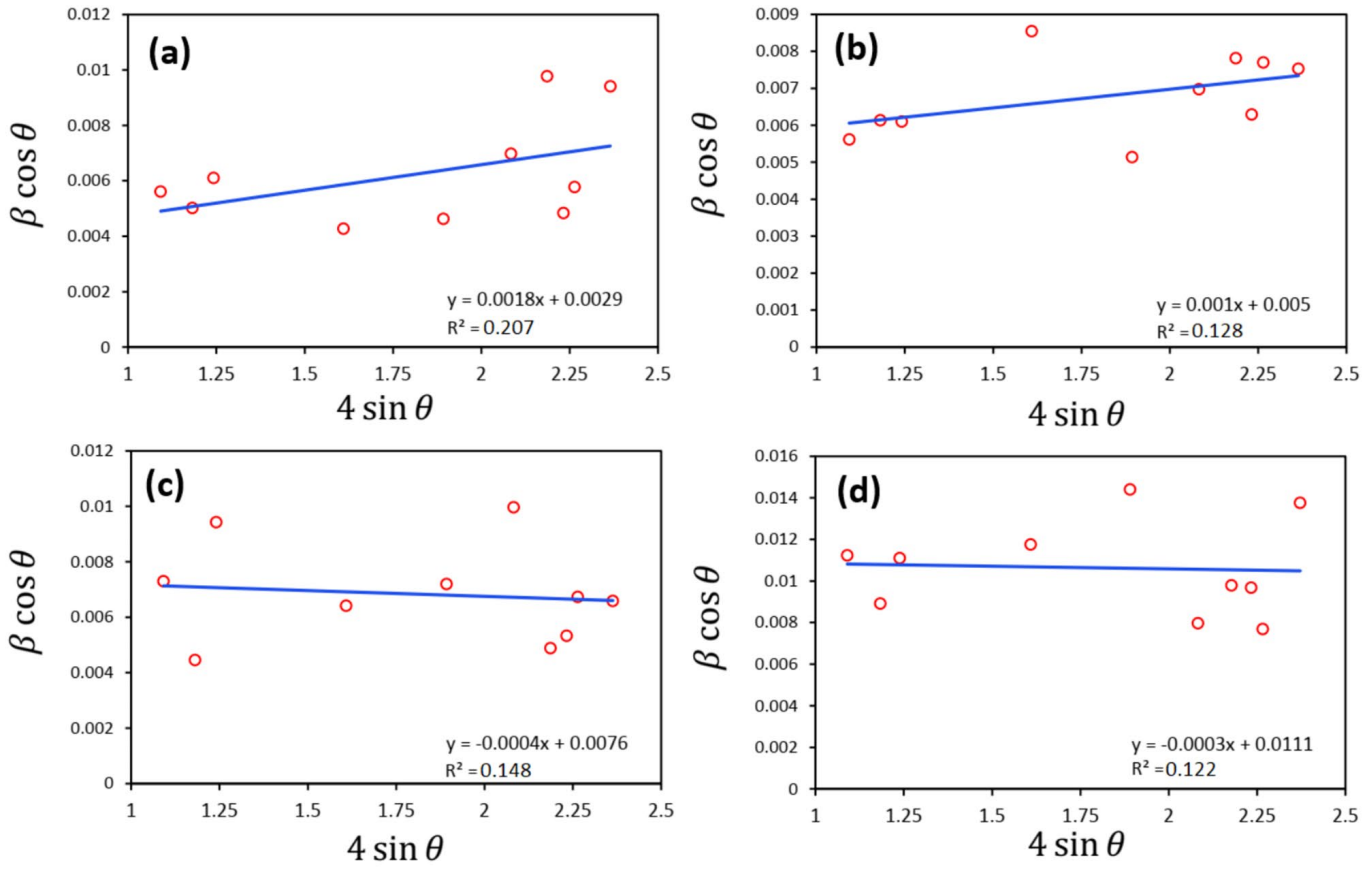
W–H plots attributed to (**a**) ZnO, (**b**) ZG3, (**c**) ZG9, and (**d**) ZG15.

The XRD data are used to draw the linearly fitted data and the results are presented if Fig. 5. The results are obtained to be 51, 29, 19, and 13 nm for ZnO, ZG3, ZG9, and ZG15, respectively. When Ga^3^⁺ ions substitute Zn^2^⁺ ions in the ZnO lattice, their smaller ionic radius (0.62 Å for Ga^3^⁺ vs. 0.74 Å for Zn^2^⁺ in tetrahedral coordination) leads to lattice contraction. This contraction reduces the lattice parameters and creates compressive strain within the crystal structure. In XRD-based strain analysis (using Williamson–Hall or similar methods), compressive strain is conventionally expressed as negative strain values, while tensile strain is expressed as positive values. Thus, the negative strain values observed after Ga doping indicate that the ZnO lattice is under compressive stress, which is consistent with the substitution of smaller cations that shrink the crystal lattice. This effect becomes more pronounced with increasing Ga concentration, as more Zn^2^⁺ sites are replaced by Ga^3^⁺ ions, leading to a progressive lattice contraction.

The Williamson–Hall plot’s indication of isotropic line broadening suggests that the crystallite domains in the material are isotropic in shape (e.g., spherical) and that micro-strain contributes to peak broadening, necessitating a method like the size-strain plot (SSP) to better decouple these effects. The SSP reduces reliance on high-angle diffraction peaks, where measurement precision is typically lower, by averaging data across reflections and assigning less weight to high-angle terms^46^. In this approach, crystallite size-related broadening is modelled as a Lorentzian function (reflecting the 1/cosθ dependence of size effects), while strain-induced broadening is treated as a Gaussian function (due to the tanθ dependence of lattice distortions), allowing more accurate separation of size (D) and strain (ε) parameters compared to traditional Williamson–Hall analysis^47^.

The size-strain plot (SSP) method is an advanced X-ray diffraction (XRD) technique used to estimate crystallite size and lattice strain simultaneously, addressing limitations of the Scherrer equation, which ignores strain, and the Williamson–Hall method, which assumes uniform strain. This approach better handles materials with anisotropic strain or non-uniform deformation compared to earlier methods, though it assumes isotropic strain and requires multiple diffraction peaks for accuracy. Key steps include measuring β for several peaks, correcting for instrumental broadening, and ensuring proper linear regression. While versatile for nanomaterials, ceramics, or deformed metals, its precision depends on peak quality, and results should be validated with microscopy (e.g., TEM) for complex systems.

We found that the peak broadening (β) in XRD arises from crystallite size (D) cusses broadening peaks (${\beta }_{size}=\frac{K\lambda }{D\mathrm{cos}\theta }$) and lattice strain induced additional broadening ${\beta }_{strain}=4\varepsilon \mathrm{tan}\theta$. For independent broadening mechanisms, the total variance (σ2) of the peak profile is the sum of individual variances, Eq. (8)^48,49^

8 $${\sigma }_{total}^{2}={\sigma }_{size}^{2}+{\sigma }_{strain}^{2}$$

Scince FWHM (β_hkl_ relates to variance (${\sigma }^{2}\propto {\beta }^{2}$) therefore we can have Eq. (9).

9 $${\beta }_{hkl}^{2}={\beta }_{size}^{2}+{\beta }_{strain}^{2}$$

Substituting the expression for β_size_ and β_strain_ from the earlier achievements size strain plot equation is derived, Eq. (10).

$${\beta }_{hkl}^{2}={\left(\frac{K\lambda }{D\mathrm{cos}\theta }\right)}^{2}+{\left(4\varepsilon \mathrm{tan}\theta \right)}^{2}={\left(\frac{K\lambda }{D\mathrm{cos}\theta }\right)}^{2}+{\left(4\varepsilon \frac{\mathrm{sin}\theta }{\mathrm{cos}\theta }\right)}^{2}\underset{}{\Rightarrow }$$

10 $${\left({\beta }_{hkl}\mathrm{cos}\theta \right)}^{2}={\left(\frac{K\lambda }{D}\right)}^{2}+{\varepsilon }^{2}{\left(4\mathrm{sin}\theta \right)}^{2}$$

#### Crystallite size of Ga-doped ZnO-NPs using SSP method

**Fig. 6:**
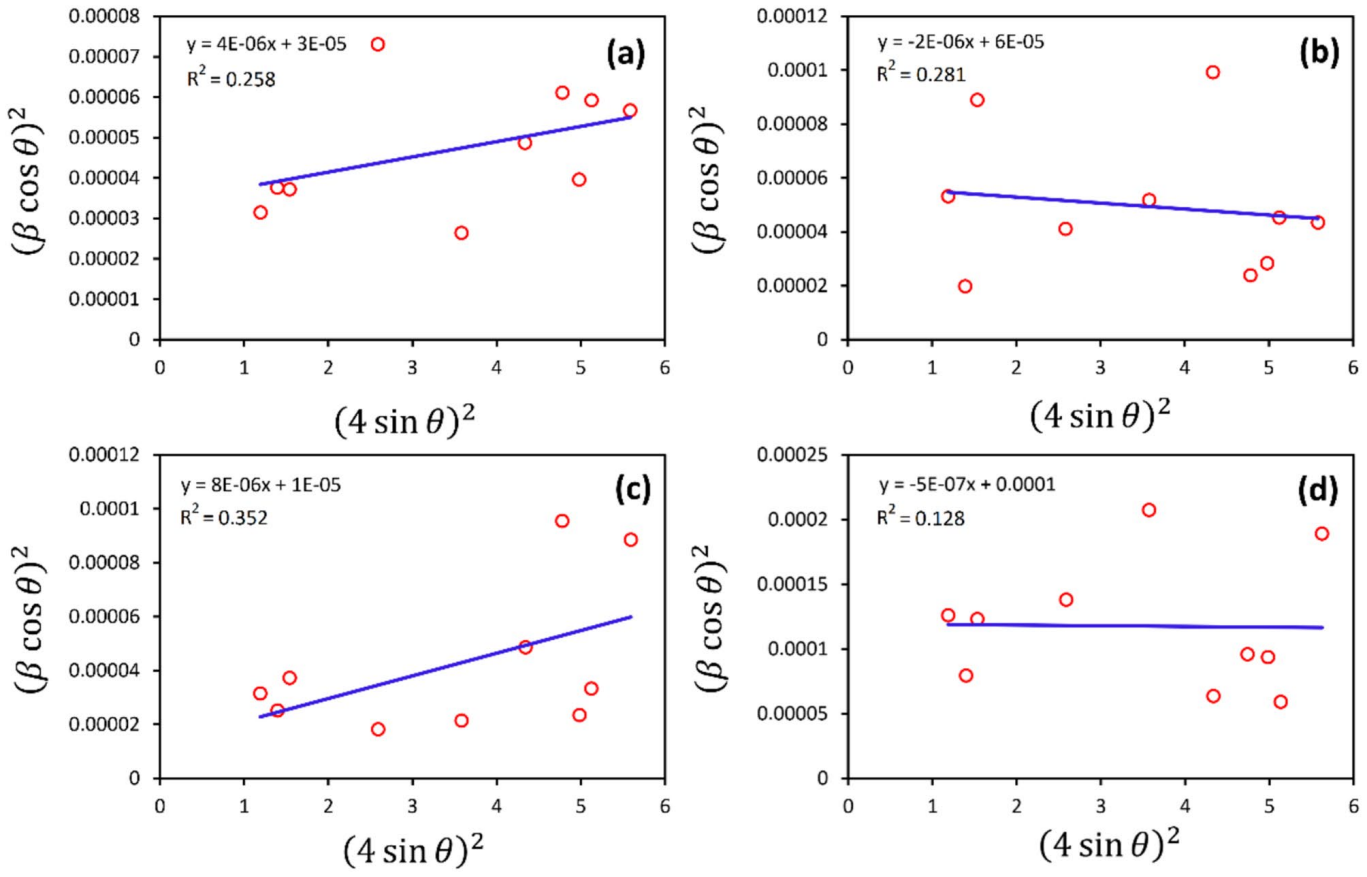
SSP plots attributed to (**a**) ZnO, (**b**) ZG3, (**c**) ZG9, and (**d**) ZG15.

By plotting ${\left({\beta }_{total}\mathrm{cos}\theta \right)}^{2}$ with respect to ${\left(4\mathrm{sin}\theta \right)}^{2}$, D and ε are are obtained from the slope and y-intersection of the linearly fitted data, respectively, Fig. 6. The results are obtained to be 47, 27, 19, and 15 nm, for ZnO, ZG3, ZG9, and ZG15, respectively.

This relation can be simplified as below:

$${\left({\beta }_{hkl}\mathrm{cos}\theta \right)}^{2}=\left(\frac{K\lambda }{D}\right){\lambda }^{2}\left(\frac{K\lambda }{D}\right)+{\varepsilon }^{2}{\left(4\mathrm{sin}\theta \right)}^{2}$$

From the Scherrer equation, Eq. (4):

$$D=\frac{K\lambda }{{\beta }_{D}\mathrm{cos}\theta }\underset{}{\Rightarrow }\frac{K}{D}=\frac{{\beta }_{D}\mathrm{cos}\theta }{\lambda }$$

where ${\beta }_{D}={\beta }_{size}$ and from Bragg equation:

$$\lambda =2d\mathit{sin}\theta \stackrel{}{\Rightarrow }2\mathrm{sin}\theta =\frac{\lambda }{d}$$

Therefore, we can write:

$${\left({\beta }_{hkl}\mathrm{cos}\theta \right)}^{2}=\frac{K}{D}{\lambda }^{2}\frac{{\beta }_{D}\mathrm{cos}\theta }{\lambda }+4{\varepsilon }^{2}\frac{{\lambda }^{2}}{{d}^{2}}$$

Multiply both side by $\frac{{d}^{2}}{{\lambda }^{2}}$

$$\frac{{\left(d{\beta }_{hkl}\mathrm{cos}\theta \right)}^{2}}{{\lambda }^{2}}=\left(\frac{K}{D}\right)\frac{{d}^{2}{\beta }_{D}\mathrm{cos}\theta }{\lambda }+{\left(2\varepsilon \right)}^{2}$$

It can be consumed that βD is a coefficient of βhkl (${\beta }_{D}=\acute{k}{\beta }_{hkl}$) therefore the above relation modified as below and modified size strain plot (MSSP) relation is derived, Eq. (11).

$$K\acute{k}{\beta }_{hkl}=K{\beta }_{hkl} and K=\raisebox{1ex}{$3$}\!\left/ \!\raisebox{-1ex}{$4$}\right.\underset{}{\Rightarrow }$$

11 $$\frac{{\left(d{\beta }_{hkl}\mathrm{cos}\theta \right)}^{2}}{{\lambda }^{2}}=\left(\frac{K}{D}\right)\frac{{d}^{2}{\beta }_{hkl}\mathrm{cos}\theta }{\lambda }+{\left(2\varepsilon \right)}^{2}$$

In this form the term $\frac{{\left(d{\beta }_{hkl}\mathrm{cos}\theta \right)}^{2}}{{\lambda }^{2}}$ is plotted with respect to $\frac{{d}^{2}{\beta }_{hkl}\mathrm{cos}\theta }{\lambda }$ and then D is estimated from the sloop and ε from the y-intersection of the linearly fitted data.

If from the beginning we ${\beta }_{D}=\acute{k}{\beta }_{hkl}$ the final relation, Eq. (11), that derived above can be obtained more simply.

$${\beta }_{hkl}^{2}={\beta }_{D}^{2}+{\beta }_{\varepsilon }^{2} \underset{}{\Rightarrow } \acute{k}{\beta }_{hkl}\frac{K\lambda }{D\mathrm{cos}\theta }+{\left(4\varepsilon \mathrm{tan}\theta \right)}^{2}$$

For the strain broadening part:

$${\left(4\varepsilon \mathrm{tan}\theta \right)}^{2}={\left(4\varepsilon \frac{\mathrm{sin}\theta }{\mathrm{cos}\theta }\right)}^{2}={\left(2\varepsilon \frac{\lambda }{d\mathrm{cos}\theta }\right)}^{2}={\left(2\varepsilon \right)}^{2}\raisebox{1ex}{${\lambda }^{2}$}\!\left/ \!\raisebox{-1ex}{${d}^{2}{\left(\mathrm{cos}\theta \right)}^{2}$}\right.$$

By substituting this relation in Eq. (9).

$${\beta }_{hkl}^{2}=\acute{k}{K\beta }_{hkl}\frac{\lambda }{D\mathrm{cos}\theta }+{\left(2\varepsilon \right)}^{2}\raisebox{1ex}{${\lambda }^{2}$}\!\left/ \!\raisebox{-1ex}{${d}^{2}{\left(\mathrm{cos}\theta \right)}^{2}$}\right.$$

Similar as mentioned earlier we consider $\acute{k}K=K\approx \frac{3}{4}$ for normal spherical particles and therefore the final SSP relation is presented as below:

12 $$\frac{{\left(d{\beta }_{hkl}\mathrm{cos}\theta \right)}^{2}}{{\lambda }^{2}}=\left(\frac{K}{D}\right)\frac{{d}^{2}{\beta }_{hkl}\mathrm{cos}\theta }{\lambda }+{\left(2\varepsilon \right)}^{2}$$

#### Crystallite size of Ga-doped ZnO-NPs using modified SSP (MSSP) method

**Fig. 7:**
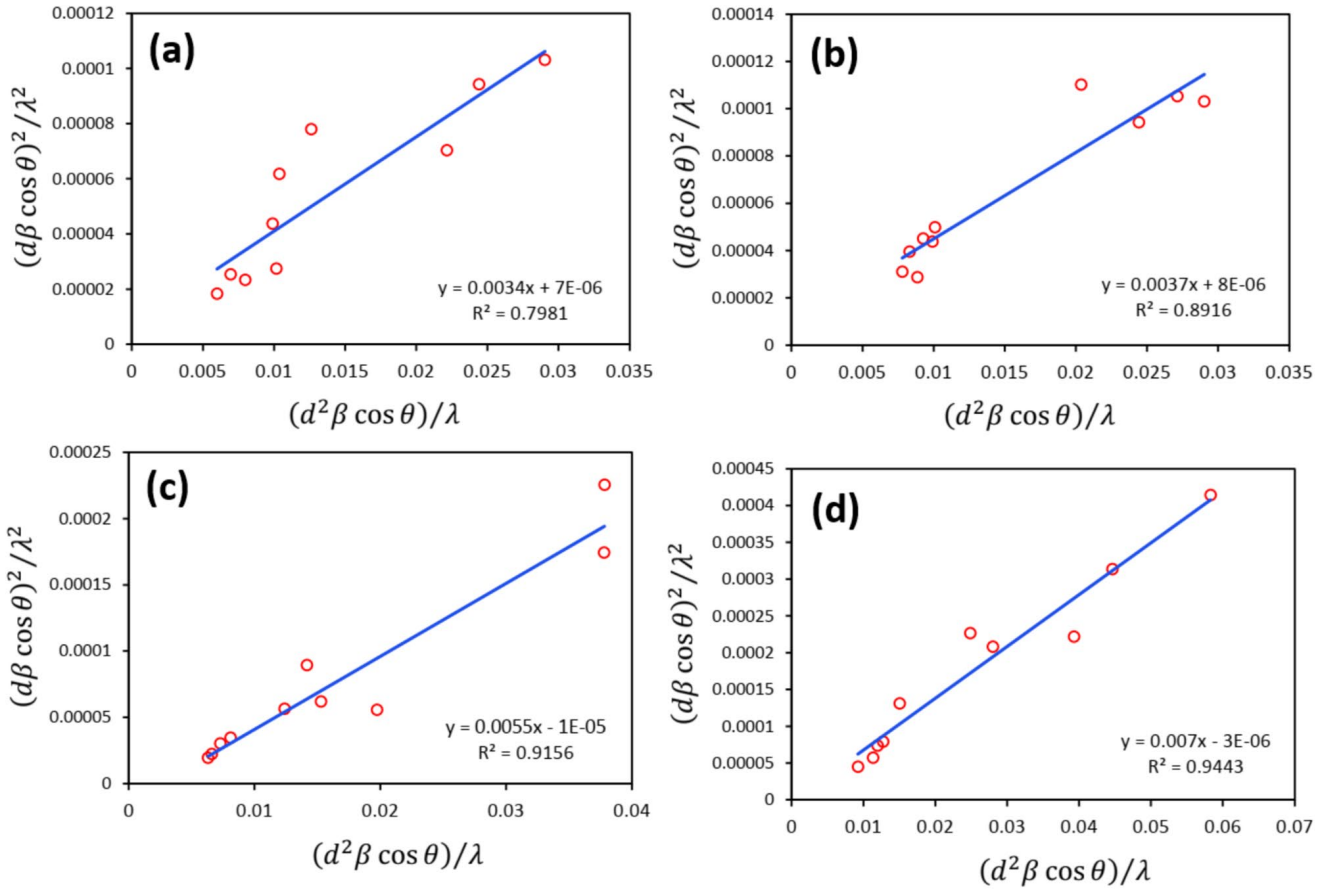
MSSP plots attributed to (**a**) ZnO, (**b**) ZG3, (**c**) ZG9, and (**d**) ZG15.

By plotting $\frac{{\left(d{\beta }_{hkl}\mathrm{cos}\theta \right)}^{2}}{{\lambda }^{2}}$ with respect to $\frac{{d}^{2}{\beta }_{hkl}\mathrm{cos}\theta }{\lambda }$, D and ε are obtained from the sloop and y-intersection of the linearly fitted data, respectively, Fig. 7. The results are obtained to be 22, 20, 14, and 11 nm, for ZnO, ZG3, ZG9, and ZG15, respectively.

### TEM observations

**Fig. 8:**
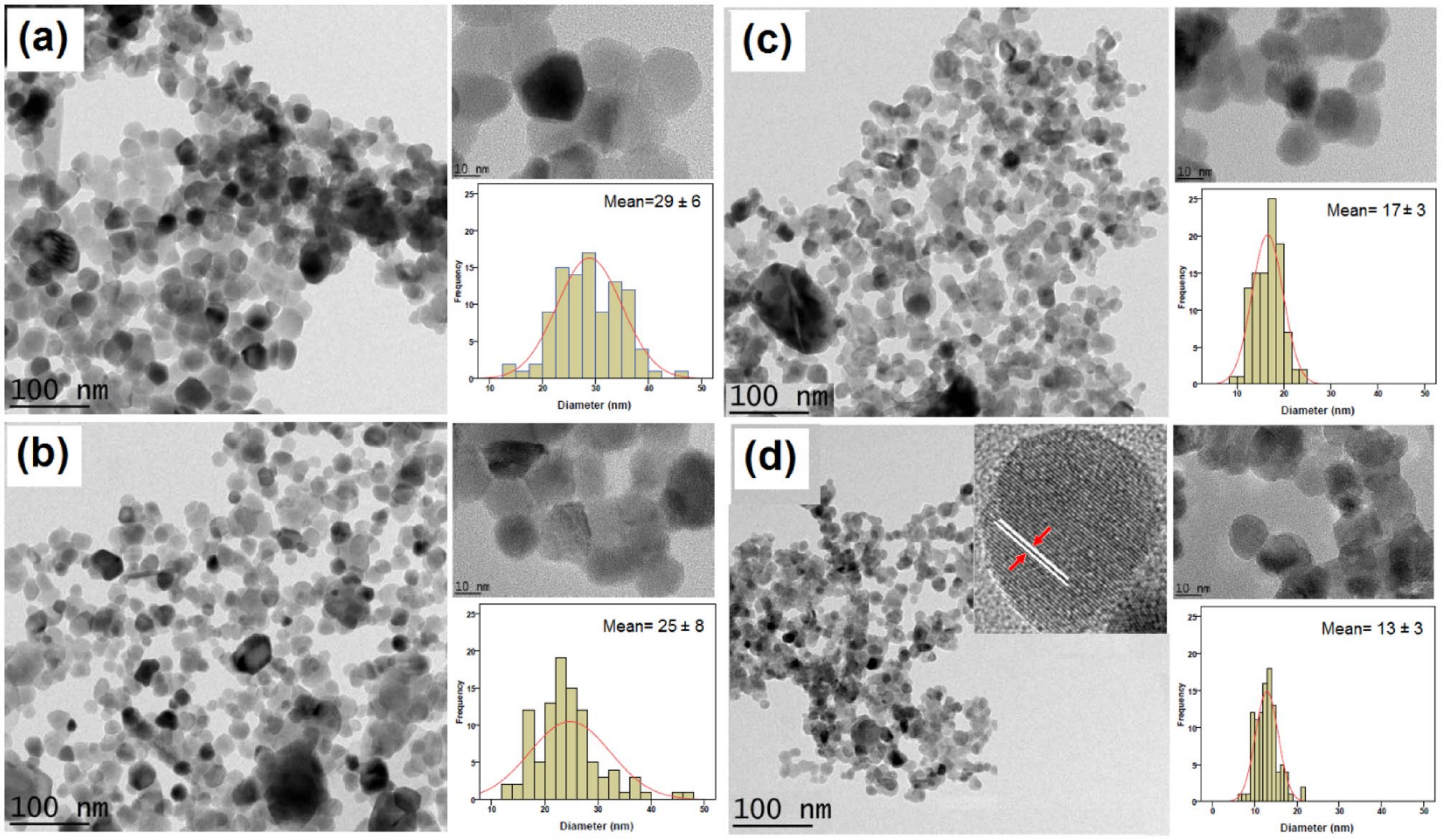
TEM micrographs attributed to (**a**) ZnO, (**b**) ZG3, (**c**) ZG9, and (**d**) ZG15.

The morphology and particle size of the synthesized samples were examined using transmission electron microscopy (TEM). Figure 8 shows TEM images of ZnO, ZG3, ZG9, and ZG15 at both low and high magnifications, along with corresponding particle size distribution histograms. A clear trend is observed where increasing dopant content leads to a reduction in average particle size, which aligns well with the XRD findings. The particles generally exhibit a spherical shape. The average particle sizes for ZnO, ZG3, ZG9, and ZG15 were approximately 29 ± 6 nm, 25 ± 8 nm, 17 ± 3 nm, and 13 ± 3 nm, respectively. A high-resolution TEM image of the ZG15 sample is presented in the inset of Fig. 8d, revealing its single-crystalline nature.

All the obtained results for the crystallite and particle sizes are presented in Table 1 to have a better vision for the comparison.

**Table 1:** The summarized results of the calculated and measured crystallite and particle sizes.

Sample	Crystallite size (nm)				Particle size (nm)
	Scherrer	W–H	SSP	MSSP	TEM
ZnO	26*	51*	47*	22	29
ZG3	24	29*	27*	20	25
ZG9	22*	19*	19*	14	17
ZG15	13	13	15*	11	13

The average crystallite size cannot be larger than particle size. Therefore the most accurate results have been obtained by MSSP method.

*Shows the obtained crystallite sizes by the other methods which are larger than particle sizes.

## Conclusion

In this study, Ga-doped ZnO nanoparticles (ZnO-NPs) were synthesized via a gelatin-assisted sol–gel method, and their structural and morphological characteristics were systematically investigated. Various X-ray diffraction (XRD) analysis techniques, including the Scherrer method, Williamson–Hall (W–H) plot, Size-Strain Plot (SSP), and modified SSP (MSSP), were employed to determine crystallite sizes with improved precision. The results revealed a clear reduction in crystallite size with increasing gallium content. Specifically, the crystallite sizes obtained using the MSSP method for the ZnO, ZG3, ZG9, and ZG15 samples were 22, 20, 14, and 11 nm, respectively. This reduction is attributed to the successful incorporation of Ga^3^⁺ ions into the ZnO lattice, inducing lattice strain and suppressing grain growth. This trend is consistent with transmission electron microscopy (TEM) observations, where the corresponding average particle sizes were 29, 25, 17, and 13 nm, respectively. The nanoparticles exhibited a predominantly spherical morphology, and high-resolution TEM images confirmed the monocrystalline nature of the ZG15 sample. Comparative analysis of the XRD and TEM results indicated that the MSSP method provided a more reliable estimation of crystallite size and structural evolution.

## Data Availability

The datasets generated and/or analysed during the current study are available in the Crystallography Open Database (COD) repository, [COD ID: 1011260, https://www.crystallography.net/cod/1011259.html].
